# Incidence and Distribution of Microfungi in a Treated Municipal Water Supply System in Sub-Tropical Australia

**DOI:** 10.3390/ijerph7041597

**Published:** 2010-04-06

**Authors:** Noel B. Sammon, Keith M. Harrower, Larelle D. Fabbro, Rob H. Reed

**Affiliations:** 1 Centre for Plant and Water Science, CQUniversity Australia, Rockhampton, Queensland, Australia; E-Mail: r.reed@cqu.edu.au; 2 Centre for Environmental Management, CQUniversity Australia, Rockhampton, Queensland, Australia; E-Mail: l.fabbro@cqu.edu.au

**Keywords:** *Aspergillus*, microfungi, drinking water, human pathogens, nosocomial mycoses, immunosuppressed

## Abstract

Drinking water quality is usually determined by its pathogenic bacterial content. However, the potential of water-borne spores as a source of nosocomial fungal infection is increasingly being recognised. This study into the incidence of microfungal contaminants in a typical Australian municipal water supply was carried out over an 18 month period. Microfungal abundance was estimated by the membrane filtration method with filters incubated on malt extract agar at 25 °C for seven days. Colony forming units were recovered from all parts of the system and these were enumerated and identified to genus level. The most commonly recovered genera were *Cladosporium*, *Penicillium*, *Aspergillus* and *Fusarium*. Nonparametric multivariate statistical analyses of the data using MDS, PCA, BEST and bubble plots were carried out with PRIMER v6 software. Positive and significant correlations were found between filamentous fungi, yeasts and bacteria. This study has demonstrated that numerous microfungal genera, including those that contain species which are opportunistic human pathogens, populate a typical treated municipal water supply in sub-tropical Australia.

## 1. Introduction

The quality of drinking water is usually determined by the level of pathogenic bacteria it contains, and the standard indicator is the number of *Escherichia coli* CFU per 100 mL of water as stated, for example, in the Australian Drinking Water Guidelines (2004) (http://www.nhmrc.gov.au/publications/synopses/_files/adwg_11_06.pdf). Many studies have been conducted into the contamination of drinking water by bacterial pathogens including enteric and aquatic bacteria. Enteric protozoa such as *Giardia* and *Cryptosporidium*, which are serious gastrointestinal human pathogens, have also received attention in the scientific literature as drinking water contaminants [1–4]. On the other hand, few studies have been carried out on microfungal contamination of municipal drinking water systems [5–17]. This may be because the only primary human pathogens are the genera *Histoplasma*, *Coccidioides*, *Blastomyces* and *Paracoccidioides* and these are not known to be waterborne. However there are a number of opportunistic human fungal pathogens which are known to be waterborne [5–17] and several studies have suggested that inhalation of water aerosols containing spores of those fungi may be the route for systemic infection in humans [18–23]. Furthermore, some fungal species isolated from water supplies are potentially allergenic or toxigenic [12], while others may produce off-tastes and odours [24].

Neither the World Health Organisation's Guidelines for Drinking Water Quality nor the Australian Drinking Water Guidelines contain standards covering microfungal contamination of drinking water supplies. Sweden is the only country known to include specific criteria for maximum numbers of microfungi in drinking water; the Swedish Drinking Water Guidelines specify 100 CFU of microfungi per 100 mL of water as the criterion [8].

The objective of our study was to determine the quantitative and qualitative characteristics of microfungi and their distribution, to assess their correlation with each other and with environmental factors in a typical municipal water supply system in sub-tropical Australia, and to provide data of value in further research into the effects of waterborne microfungi on human health under those conditions. The study focused on the microfungal population of the water treatment and distribution system. However, data were also collected on the bacterial population to assess the relationships between these two groups of microorganisms.

## 2. Materials and Methods

### 2.1. Water Supply System

The single source of raw water for this system is a riverine impoundment with a capacity of *ca*. 80,000 megalitres and a catchment area of *ca*. 139,000 km^2^. The raw water is treated by coagulation/flocculation with polyaluminium chlorhydrate and a polymer followed by sedimentation, sand filtration, and disinfection with chlorine to *ca*. 0.60 ppm before leaving the single treatment plant. The treated water is then pumped to 12 roofed, above-ground storage reservoirs throughout the city and is distributed from these reservoirs to consumers through reticulation mains. In several localised areas, water is delivered to consumers directly from the trunk mains. Some of the reservoirs are rechlorinated, either automatically or manually on a regular basis, with the aim of maintaining free chlorine at *ca*. 0.60 ppm in the reservoirs and *ca*. 0.20 ppm at the extremities of the mains distribution system. During the course of this study two of the reservoirs were not rechlorinated.

### 2.2. Sample Collection

Samples were drawn monthly during the period May 2007 to October 2008 from standpipes at nine mains sites (designated A, B, C, D, E, H, J, K, Z), directly from six reservoirs (designated 1–6), and from four sites at the treatment plant (R = raw water, F = flocculated water, S = sand-filtered water, T = treated water). The taps on mains standpipes were sprayed with 70% ethanol and were then flushed at maximum capacity for three minutes immediately prior to sample collection. At three of the reservoirs, samples were collected from off-take taps at the reservoirs and these were also disinfected and then flushed for three minutes prior to sample collection. Samples from the other three reservoirs were collected directly from the water body in the reservoirs. Triplicate 500 mL samples from each site were collected in sterile polypropylene, wide-mouth screw-top bottles (Nalgene™); two replicates for mycological analysis and one for bacteriological analysis. Each sample bottle was sealed immediately after the sample was collected and was then placed in a pre-cooled insulated container for transport to the laboratory. Streptomycin was added at 100 mg L^−1^ to each mycological sample bottle prior to sampling to inactivate bacteria. At the laboratory, 60 mg of sodium thiosulphate was added to all samples to inactivate any residual free chlorine. Samples were processed within 8 h of collection.

### 2.3. Physico-chemical Parameters

Physico-chemical properties of the water at each site were recorded monthly as the mean of three readings taken at three minute intervals with a YSI 6600M multi-parameter water meter. Variables included temperature (°C), pH, conductivity (μS cm^−1^), oxidation-reduction potential (mV) (ORP), dissolved oxygen concentration (mg L^−1^) and turbidity (NTU). Free chlorine (ppm) was measured with a Eutech C201 chlorine meter.

### 2.4. Microfungal Analyses

Microfungal loads were estimated by membrane filtration (Millipore™ 0.45 μm pore size, 47 mm diam) of each 500 mL sample. Filter membranes were placed on culture plates of malt extract agar (MEAC) (malt extract 10 g, glucose 10 g, bacteriological agar 10 g, peptone 0.5 g, 500 mL reverse osmosis water and chloramphenicol 50 mg), and these were incubated at 25 °C in the dark. This is known from our previous work to be the approximate median water temperature over a 12 month period in the sub-tropical city where the study was conducted. Use of this incubation temperature was subsequently justified by the actual water temperatures which ranged from 17 °C to 30 °C with a median of 23.5 °C over the study period. The purpose was to isolate those genera which would germinate and grow within the range of temperatures experienced in this climate since it would be those genera most likely to potentially cause human health problems. The genera, such as *Aspergillus*, which are suspected aetiological agents of waterborne nosocomial mycoses are known to germinate over a range of conditions and grow well under the conditions used in this study. Preliminary tests on a number of different media showed that MEAC was an effective non-selective medium for recovery of a wide range of microfungi from different genera. Where a high fungal load was expected, the 500 mL water samples were filtered in smaller aliquots to facilitate subsequent counting of CFU and the counts were aggregated. Because of the high turbidity and high microbial population of the raw water, serial decimal dilutions of the raw water samples were performed to 10^−3^ and these were used to estimate microfungal loads of those samples. Yeast colonies and filamentous fungal colonies were counted under a Wild M3Z stereomicroscope over seven days. Identification of filamentous microfungi to genus level was made in accordance with Standard Methods for the Examination of Water and Wastewater [25] by microscopic examination of their reproductive structures and spores under a Leica DMLB light microscope, and by reference to Carmichael *et al*. [26], Ellis [27,28], and Kendrick and Carmichael [29]. For yeasts, 30 randomly selected colonies recovered from the reservoirs and mains were identified by a specialist pathology laboratory (Sullivan Nicolaides Pathology Laboratories, Rockhampton, Australia).

### 2.5. Bacteriological Analyses

Bacterial loads were estimated by membrane filtration (0.45 μm pore size). Filters were incubated on plate count agar to which 100 mg L^−1^ of nystatin had been added aseptically after autoclaving to inhibit microfungal growth. Culture plates were surface-dried in a biological safety cabinet for 2 h prior to use to restrict the spread of motile colonies. Bacterial colonies were counted under a Wild M3Z stereomicroscope after two days of incubation.

### 2.6. Statistical Analyses

Multivariate analyses of the data were carried out using cluster analysis, non-metric multi-dimensional scaling (MDS), principal components analysis (PCA), and the ‘BEST’ routine in PRIMER v6 statistical software (Plymouth Marine Laboratory, Plymouth UK). Bubble plots of the biological data were superimposed on the environmental PCA ordinations to graphically demonstrate the relationships between the two sets of data. SPSS Statistics 17.0 software (SPSS Australasia Pty Ltd, Sydney, Australia) was used for bivariate correlation analyses and resulting graphs were produced with SigmaPlot software (Systat Software Inc., Chicago IL).

The sampling sites were divided into three site groups; mains, reservoirs, and treatment plant. Analyses of the data for these groups were carried out separately. The biological data for each site group were divided into three groups; filamentous fungi, yeasts and yeast-like fungi, and bacteria. The samples for one month from two of the treatment plant sites were excluded because of missing data. Sixteen months of complete samples for 17 sites and 15 months of complete samples for two sites were included in the analyses. This resulted in a slightly unbalanced design but such designs are handled adequately by PRIMER v6 [30].

Turbidity was excluded from the group analyses because the mean values of that variable for all sites, except the raw water source, were very low at ≤1 NTU. However, because of the high turbidity of the raw water, a separate analysis of the raw water samples was conducted to determine any relationship between turbidity and microbial counts.

For each site group, the biological data were square-root transformed, a triangular resemblance matrix of between-sample similarities based on the Bray-Curtis coefficient was computed, and a MDS ordination of the transformed data was plotted. A draftsman plot of the environmental data for each site group was used to determine whether or not, and to what degree, transformation of one or more of those variables was desirable. The transformed data were then normalised, a triangular resemblance matrix of between-sample similarities based on Euclidian distance was computed, and the among-sample relationships were displayed on a MDS ordination. A PCA was also run on the correlation matrix (*i.e*., the transformed, normalised data). The MDS and PCA ordinations were compared and found to be very similar in each case, both being based on Euclidian distance measures. The PCA ordination with vectors displayed was therefore triplicated, and a bubble plot of each of the three biological groups was superimposed on each of the triplicate ordinations to graphically show any correlations between the biological data and the environmental variables.

The BEST routine was then run, with 50 restarts, to determine the best match between the multivariate among-sample patterns of the biological data and the environmental variables. The parameters used were BIOENV, the transformed normalised environmental data, all environmental variables, the transformed resemblance biological data, and Spearman rank correlation. Finally, the Permutation feature of BEST was used to test the null hypothesis ‘there is no agreement in the multivariate pattern of the two data sets’ using 999 permutations and a significance level of 0.001.

## 3. Results

### 3.1. Microfungal Enumeration and Identification

**Figure 1.**
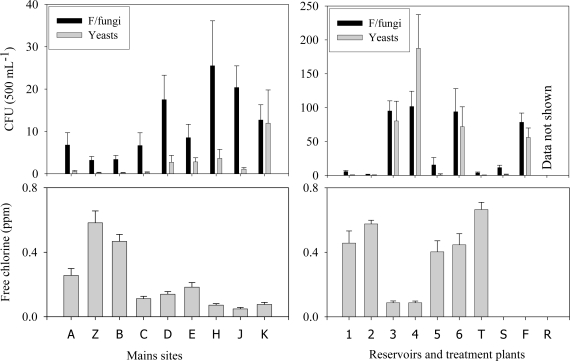
Means of filamentous fungi and yeasts recovered from reservoir and treatment plant sites compared with mean free chlorine concentrations. Error bars = standard error.

Sixty two genera of identified filamentous microfungi were recovered from the reservoirs, mains, and the treated water at the treatment plant during the period May 2007 to February 2008. Only 11 of those genera each accounted for 1% or more of the filamentous microfungal CFU recovered in any of the site groups (Table 1). Microfungi and bacteria were recovered from all parts of the water treatment and distribution systems but there was considerable variation in frequencies between sites. Filamentous fungi and yeast numbers were higher at sites where free chlorine values generally were low (Figure 1). There was a steep downward gradient in microfungal and yeast frequencies from the raw water source to the final treated product at the treatment plant.

The 30 randomly selected yeast/yeast-like colonies were identified as 18 *Aureobasidium* spp., four *Rhodotorula* spp., seven *Lecythophora* spp., and one *Cryptococcus laurentii*.

### 3.2. Statistical Analyses

Comparison of the MDS and PCA ordinations of environmental data for each site group, both based on Euclidian distance measures, showed they were very similar in each case. Measured stress levels for the 2-dimensional (2-d) MDS (biological) and 2-d MDS (environmental) were all within acceptable levels (Table 2) [30].

**Figure 2.**
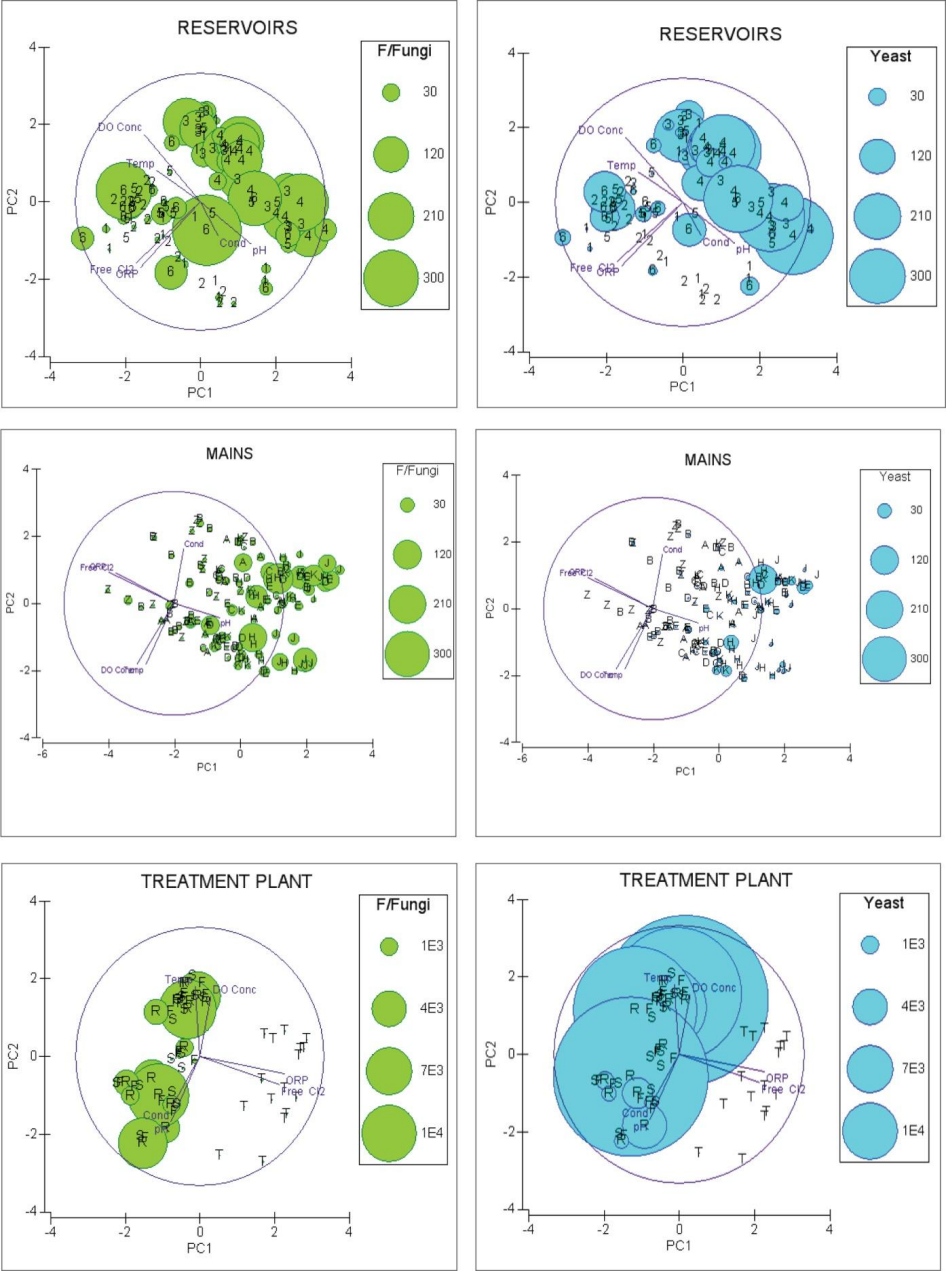
PCA ordinations of environmental data superimposed with bubble plots of estimated filamentous microfungi and yeast/yeast-like microfungi. The alpha and numeric labels signify individual sites and their monthly sample data.

The Eigenvalues for all groups showed that, in each case, a 2-d PCA gave a reasonably good description of the environmental structure in high dimensional space, with PC1 and PC2 accounting for most of the variability in the mains (56.5%), reservoirs (62.1%) and treatment plant (61.6%). For each of the three site groups, PC1, PC2, and PC3 together accounted for ≥75% of the variability between variables (Table 3). PCA ordinations of the PC1 and PC2 environmental structures for each of the three site groups, with vectors displayed, are in Figure 2. The lengths of the vectors on the 2-d PCA ordinations reflect the importance of the contributions made by the variables to the first two axes.

A two-tailed Spearman’s Rho correlation analysis at a significance level of 0.01, carried out on the all-sites data, was based on filamentous fungi (mean = 208, SD = 1,189), yeasts (mean = 1,293, SD = 8,494), and bacteria (mean = 19,316, SD = 81,665). The analysis showed positive and significant correlations between the frequencies of filamentous fungi and yeasts (ρ_(299)_ = 0.723, *P* < 0.01); between filamentous fungi and bacteria (ρ_(299)_ = 0.533, *P* < 0.01); and between yeasts and bacteria (ρ_(299)_ = 0.402, *P* < 0.01).

High numbers of filamentous fungi and yeasts were negatively correlated with low free chlorine and ORP values. The means of filamentous microfungi and yeasts/yeast-like microfungi recovered per 500 mL, and mean free chlorine concentrations recorded at each sampling site during the period July 2007 to October 2008 are in Figure 1. The raw water was excluded from this graph because of the very high frequencies and large variations at that site.

Bubble plots of filamentous fungi, yeasts, and bacteria superimposed on the environmental PCA of treatment plant data including turbidity (not illustrated), indicated that turbidity was positively correlated with high yeast and bacterial counts, but there was little correlation between turbidity and filamentous fungi. Correlation analyses using SPSS Statistics 17.0 showed that yeasts/yeast-like microfungi was the only biological group to show a significant and positive correlation with turbidity (ρ_(15)_ = 0.792, *P* < 0.01, mean = 25,328, SD = 9,734).

**Figure 3.**
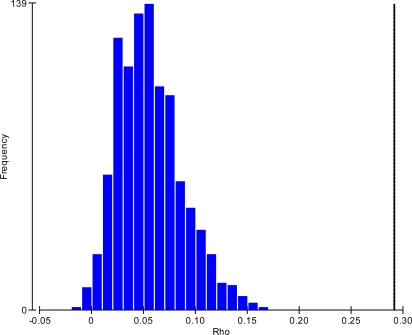
Histogram of the null hypothesis distribution of the test statistic using reservoirs data and showing real ρ as a vertical line at 0.291. 999 permutations, α = 0.001.

For each of the three site groups, the BEST analyses and null hypothesis test histograms all showed clearly that the null hypothesis ‘there is no agreement in the multivariate pattern of the two data sets’ should be rejected. The histogram for the reservoirs sites is illustrated in Figure 3. The four combinations of environmental variables with the highest rank correlation coefficients (ρ) in each of the three groups are listed in Table 2. In all cases ρ was optimised for free chlorine and the second highest ranking was for free chlorine and ORP thus confirming that these two variables best ‘explain’ the microbiological assemblage structure. The BEST analyses supported the PCA and bubble plots. Either or both of these environmental variables featured in all of the four highest rankings in each group.

## 4. Discussion

Although 62 genera of filamentous microfungi were recovered and identified during the course of this study, only 11 of them accounted for ≥1% of the total from any one site group, and there was a fairly high percentage (*ca*. 19%) of unidentified, asporogenous colonies (Table 1). It is recognised that the asporogenous colonies may have required specific media and/or conditions, or longer time frames in which to sporulate. This was a baseline study aimed at gaining an insight into whether or not microfungi were present in the water supply system, their incidence, and an overview of the community’s composition rather than precise identification of all microfungi recovered. Similar considerations applied to identification to genus level rather than to species level.

Microfungi and bacteria were recovered from all parts of the water treatment and distribution systems but they were more abundant where free chlorine concentrations were non-existent or very low. Zero to very low numbers of microfungi and bacteria were recovered from the treated water, as it left the treatment plant. Reservoirs such as R2 which were automatically rechlorinated to approximately the same concentration as the treated water also showed correspondingly low numbers. The highest recoveries were from two reservoirs where no supplementary chlorination was applied (Figure 1). In a separate study, we demonstrated that the Australian green tree frog, *Litoria caerulea*, which inhabits these reservoirs, is an important contributor of microfungal and *E. coli* contamination of post-treatment drinking water. *L. caerulea* excreta collected within the reservoirs were all found to contain *E. coli* with cell counts as high as 2.89 × 10^8^ g^−1^ wet weight mass [31]. These findings demonstrate that recontamination does occur post-treatment and, in order to prevent it, supplementary chlorination of all water reservoirs should be routinely carried out so that a free chlorine concentration of at least 0.60 ppm is maintained.

The steep downward gradient in microfungal frequencies from the raw water source to the final treated product at the treatment plant (Figure 1) shows that these microorganisms are very effectively removed by sequential chemical coagulation, sedimentation, and sand filtration, even before disinfection is applied. This observation is supported by the treatment plant PCA ordination and bubble plots (Figure 2) and also confirms the findings of Niemi, Knuth and Lundstrom [14] who reported that the incidence of fungi in water treated by chemical coagulation was lower than in water treated by sand filtration alone.

Analysis of the environmental variables of the reservoirs samples showed that free chlorine, ORP and dissolved oxygen had the most sizeable coefficients (Eigenvectors) on PC1, and the coordinates of these three variables were very closely linked. Free chlorine and ORP maintained their position on PC2 but dissolved oxygen showed a marked decrease in its contribution to that axis. None of those three variables had any marked effect on PC3. The principal component on PC3 was conductivity which had a high coefficient of 0.805 in the positive direction.

In the mains samples, free chlorine and ORP, both with negative values similar to each other, were again closely associated and were important contributors to PC1 but they both decreased markedly on PC2 and had little influence on PC3. Temperature and dissolved oxygen, both with negative values, were *closely* associated with each other and increased markedly from PC1 to PC2 to become the most important contributors to the PC2 axis although free chlorine and ORP were still important contributors.

The *PCA* of the treatment plant sites was characterised by the very large effect of free chlorine and ORP on PC1 due to the high free chlorine concentration in the treated water and zero free chlorine at the other three sites. Those two variables decreased markedly to negative values on PC2 and became positively correlated with pH and conductivity but negatively correlated with temperature and dissolved oxygen. Conductivity and dissolved oxygen were the principal components of PC3 with negative coefficient values, and temperature showed a positive relationship to those two variables.

Superimposition of bubble plots of the filamentous fungi and yeast data on the PCA ordinations of the physico-chemical properties for the three site groups showed a clear pattern of greatly reduced numbers of microfungi with increasing free chlorine and ORP values. This pattern was particularly evident in the treatment plant sites where three of the sites were not chlorinated and one (the final *treated* product) was chlorinated with mean free chlorine of 0.67 ppm. Conversely in the mains, where free chlorine and ORP values were relatively low, the microfungal abundance increased. The reservoirs showed a similar pattern to that of the mains, except in respect of several outliers of filamentous fungi and yeasts associated with reservoir R6 which experienced fluctuating concentrations of free chlorine during the study period. This contrasts with the finding of Nagy and Olsen [13] who reported that there was no correlation between free chlorine concentrations and filamentous microfungal frequencies in their study. They also reported a significant positive correlation between turbidity and filamentous fungi but that relationship was not found in our study. However, the positive and significant correlation between turbidity and yeasts in the raw water found in our study agrees with the results reported by Alvarez [5].

The present study found significant and positive correlations between the frequencies of filamentous microfungi, yeasts, and bacteria when the combined data from all sample sites were analysed. Arvanitidou *et al*. [20] and West [16] also reported a positive correlation between filamentous fungi and bacteria. By contrast Nagy and Olsen [13] reported that there was no correlation between filamentous fungal and bacterial frequencies in their study. They stated they had expected to find a significant negative correlation because of the similar nutritional requirements of the two organisms and their antagonistic behaviour toward each other. However, that hypothesis relies on filamentous fungi actively growing in competition with bacteria in the same environment. Research conducted by the present authors indicates that filamentous microfungi are not growing to any noticeable extent within the water supply *system* under study, and that filamentous microfungi recovered from that system largely result from planktonic spores introduced from outside the system (N.B. Sammon, K.M. Harrower, L.D. Fabbro and R.H. Reed, unpublished data). The observed differences in the abovementioned studies may be due to a range of factors including raw water source, treatment processes and distribution system characteristics. In some systems, filamentous fungi may be mainly introduced post treatment, from outside sources (such as in the case of this study, with Australian green frogs inhabiting some reticulation reservoirs). In other systems, filamentous fungi may be introduced post treatment from outside sources as well as from populations growing within biofilms or other materials (such as pipe joints and seals) in the distribution system [24,32–34].

It is notable that *Aspergillus* was among the genera most commonly recovered from all parts of the water supply system involved in this study. Some species within that genus, particularly *A. fumigatus* and to a lesser extent *A. niger*, are opportunistic human pathogens which cause invasive aspergillosis. Concern has been raised in recent years that hospital water supplies, which are usually sourced from municipal systems, may be a route of nosocomial infection by *Aspergillus* and other microfungal genera [18–23]. Previously it was thought that such aetiological agents of nosocomial infections were airborne only [19]. It is also noteworthy that some of the other microfungi and yeasts which were most commonly recovered in the present study have also been shown to be opportunistic human pathogens, either superficial or systemic [35], thus supporting the possibility that waterborne microfungi may be a risk to human health. Rippon [35] noted that opportunistic fungal infections were formerly quite rare but in recent years have become much more common and of great medical significance. Those in the community at most risk are patients who are immuno-compromised, either through the effects of disease such as AIDS or through suppression of the immune system by medical intervention. There is an increasing trend towards shorter hospitalisation periods with subsequent on-going therapies administered after discharge of such patients. This raises the possibility of an increasing incidence of invasive mycoses contracted at home through opportunistic fungal pathogens carried in the water supply.

The results of this Australian study will assist in understanding microfungal contamination of municipal water supply systems but further taxonomic work on species identification of potential pathogens is warranted. The results also show that coagulation/flocculation, sand filtration, and chlorination to 0.60 ppm is very effective in removing microfungal contaminants from the raw water but that re-contamination will occur if supplementary chlorination of all water service reservoirs is not routinely carried out. Oxidation-reduction potential should also be measured at frequent and regular intervals as a good indication of the level and effectiveness of free chlorine in the system.

## Figures and Tables

**Table 1.** Microfungi recovered and identified during the period May 2007 to February 2008 inclusive.

	Mains		Reservoirs		Treated water ex Treatment Plant	

	CFU	%	CFU	%	CFU	%
**Genus**						
*Cladosporium*	333	37.8	967	44.4	13	37.1
*Penicillium*	81	9.2	315	14.5	0	0.0
*Aspergillus*	36	4.1	119	5.5	2	5.7
*Trichoderma*	11	1.2	18	0.8	0	0.0
*Fusarium*	27	3.1	43	2.0	5	14.3
*Pithomyces*	28	3.2	39	1.8	0	0.0
*Alternaria*	22	2.5	34	1.6	0	0.0
*Paecilomyces*	29	3.3	31	1.4	0	0.0
*Acremonium*	44	5.0	8	0.4	0	0.0
*Epicoccum*	8	0.9	26	1.2	0	0.0
*Curvularia*	6	0.7	30	1.4	0	0.0
Other genera < 1%	120	13.6	103	4.7	3	8.6
Asporogenous colonies	136	15.4	443	20.4	12	34.3

Total filamentous fungi	881	100	2176	100	35	100
Yeasts/yeast-like fungi	151			2,699		

Total microfungi	1,032			4,875		

**Table 2.** Results of MDS and BEST analyses of environmental data for the three site groups.

MDS Biological			MDS Environmental			BEST analyses		

Stress			Stress			No. of var.	Corr.	Selection (variables)
Min.		Measured	Min.		Measured			
MAINS								
0.01	3-d	0.00	0.01	3-d	0.11	1	0.476	6
	2-d	0.01		2-d	0.20	2	0.428	5, 6
						3	0.426	1, 5, 6
						2	0.399	1, 6
						Significance level = 0.001 999 permutations		
RESERVOIRS								
0.01	3-d	0.00	0.01	3-d	0.10	1	0.291	6
	2-d	0.01		2-d	0.20	2	0.265	5, 6
						3	0.235	2, 5, 6
						4	0.229	1, 2, 5, 6
						Significance level = 0.001 999 permutations		
TREATMENT PLANT								
0.01	3-d	0.04	0.01	3-d	0.08	1	0.725	6
	2-d	0.06		2-d	0.17	2	0.650	5, 6
						1	0.636	5
						3	0.634	3, 5, 6
						Significance level = 0.001 999 permutations		

**Table 3.** Results of Principal Components analyses of environmental data for the three site groups.

Principal components analyses										

PC No	Eigen values	% var	Cum % var	Variables	Eigenvectors					
						PC1	PC2	PC3	PC4	PC5
MAINS										
1	2.06	34.3	34.3	1	Temp	−0.254	−0.546	−0.129	0.668	−0.418
2	1.33	22.2	56.5	2	Cond	0.088	0.484	0.485	0.667	0.234
3	1.14	19.0	75.5	3	DO conc	−0.337	−0.546	0.410	−0.105	0.629
4	0.847	14.1	89.6	4	pH	0.419	−0.129	−0.623	0.237	0.495
5	0.416	6.9	96.6	5	ORP	−0.598	0.280	−0.191	−0.113	−0.102
				6	Free Cl_2_	−0.530	0.273	−0.395	0.172	0.346
RESERVOIRS										
1	2.24	37.3	37.3	1	Temp	−0.349	0.244	−0.254	−0.768	0.401
2	1.49	24.8	62.1	2	Cond	0.141	−0.266	0.805	−0.486	−0.146
3	0.972	16.2	78.4	3	DO conc	−0.450	0.523	0.102	−0.020	−0.704
4	0.905	15.1	93.4	4	pH	0.403	−0.327	−0.521	−0.388	−0.542
5	0.256	4.3	97.7	5	ORP	−0.481	−0.514	−0.042	0.149	0.043
				6	Free Cl_2_	−0.512	−0.474	−0.064	−0.038	−0.162
TREATMENT PLANT										
1	1.98	33.0	33.0	1	Temp	−0.038	0.544	−0.419	0.642	−0.339
2	1.72	28.6	61.6	2	Cond	−0.231	−0.404	−0.566	−0.340	−0.562
3	1.27	21.2	82.8	3	DO conc	0.089	0.465	−0.547	−0.492	0.484
4	0.517	8.6	91.4	4	pH	−0.233	−0.508	−0.386	0.465	0.568
5	0.46	7.7	99.1	5	ORP	0.685	−0.135	−0.061	0.121	0.027
				6	Free Cl_2_	0.643	−0.219	−0.227	0.024	−0.112
